# Iodine-125 Seeds Combined With Biliary Stent Placement Versus Stent Placement Alone For Unresectable Malignant Biliary Obstruction: A Meta-Analysis Of Randomized Controlled Trials

**DOI:** 10.7150/jca.49663

**Published:** 2021-01-01

**Authors:** Yucheng Xiang, Sinan Lu, Yufeng Li, Zhenghao Liu, Weilin Wang

**Affiliations:** 1 Department of Hepatobiliary and Pancreatic Surgery, The Second Affiliated Hospital, Zhejiang University School of Medicine, Hangzhou, Zhejiang 310009.; 2 Key Laboratory of Precision Diagnosis and Treatment for Hepatobiliary and Pancreatic Tumor of Zhejiang Province, Hangzhou, Zhejiang 310009.; 3 Clinical Medicine Innovation Center of Precision Diagnosis and Treatment for Hepatobiliary and Pancreatic Disease of Zhejiang University, Hangzhou, Zhejiang 310009.; 4 Clinical Research Center of Hepatobiliary and Pancreatic diseases of Zhejiang Province, Hangzhou, Zhejiang 310009.

**Keywords:** malignant biliary obstruction, ^125^I seed, biliary stent, stent patency, survival.

## Abstract

**Background and Aims:** Malignant biliary obstruction is always caused by tumors which are unresectable so that palliative stent placement is conducted for drainage of bile duct tree. Recently, irradiation stent with ^125^I seeds has been used to improve the stent patency and survival time of patients. We conducted this meta-analysis to evaluate the therapeutic efficacy and safety of biliary stent placement with^ 125^I seeds compared with stent placement alone in patients with malignant biliary obstruction.

**Methods:** We searched Pubmed, Web of Science, ClinicalTrials.gov, Cochrane Library, Embase and CNKI databases for all relevant studies up to 1 May 2020. Patient survival, stent patency, and adverse events were the primary outcome measured. Also, Review Manager 5.3 and Stata/SE15.0 were used to perform the analysis.

**Results:** Eleven randomized controlled trials with a total of 767 patients were included for meta-analysis. Stent combined with ^125^I seeds showed lower risk of stent occlusion at 3 month (Odds Ratios(OR) = 0.15; 95%CI: 0.05-0.49, *P* =0.002), 6 month (OR = 0.18; 95%CI: 0.08-0.44, *P* = 0.0001), 9 month (OR = 0.10; 95%CI: 0.05-0.20, *P* < 0.00001) and 1 year (OR = 0.15; 95%CI: 0.07-0.31, *P* < 0.00001) and better mean survival (MD = 125days; 95% CI 91-159 days; P < 0.00001) compared with stent placement alone. Also, reconstructed Kaplan-Meier data demonstrated improved survival in patients treated with stent plus ^125^I seeds (hazard ratio(HR)= 1.886; 95% CI: 1.609 to 2.210; P < 0.0001) Moreover, our analysis did not show significant difference between the two groups about the risk of adverse events including abdominal pain, hemobilia, pancreatitis, cholangitis and cholecystitis.

**Conclusion:** ^125^I seeds combined with stent demonstrated superior stent patency and improved survival time compared to stent alone with acceptable complications.

## Introduction

Malignant biliary obstruction (MBO), which means stenosis and blockage of extrahepatic or intrahepatic bile ducts, is generally caused by various cancers including cholangiocarcinoma, pancreatic cancer, gallbladder cancer and cancer metastasis 1. Due to the fact that its clinical performance is silent and unobvious, MBO is always diagnosed at an advanced stage when painless obstructive jaundice occurs. As a result, only 10%-20% of patients are resectable 2, 3. Therefore, palliative therapy with stent implantation has been widely accepted and used for several decades. Clinically, covered or uncovered self-expanding metal stents(SEMSs) and plastic stents have been used via Percutaneous Transhepatic Cholangiography (PTC) or ERCP (Endoscopic retrograde Cholangiopancreatography) and the patency time of SEMSs is better than plastic stents about 3-4 months^4^. In addition, covered SEMS is superior to uncovered SEMS in terms of stent patency but it may influence the drainage of bile, causing some other complications and it may increase the rate of stent migration 5-8. However, stent itself has no therapeutic effect on tumors, which always grow into the lumen through the stent mesh, resulting in occlusion of the stent. In addition, epithelial hyperplasia, biofilm deposition, biliary sludge, and formation of granulation tissue may cause stent occlusion as well 9, 10. So, maybe stent placement alone was not enough for patients with MBO, and stent placement combined with inner or outside radiotherapy maybe a promising treatment option. Sofi et al. 11 showed that Radiofrequency Ablation (RFA)plus stent placement was superior to stent placement alone with regard to stent patency and overall survival of patients with malignant biliary obstruction. Guo et al. [12]were the first to use ^125^I seeds with SEMS to treat advanced esophageal cancer and they showed that patients had better swallowing function and improved median survival compared with patients who received stent placement alone. Iodine-125(^125^I) seeds as a persistent radiation material can directly cause damage of the DNA double helix to inhibit the replication of tumor cells and induce apoptosis 13. Furthermore, implantation of ^125^I seeds may cause CD3^+^ and CD4^+^ cell activation and trigger antitumor immune responses^14^. Actually, previous meta-analysis 15 has compared ^125^I seeds plus stent placement with stent placement alone, but it only included five RCTs and seven nonrandomized trials, which may make it not evident enough. Moreover, significant heterogeneity of mean survival was found and no further possible reasons or influential factors were analyzed. Recently, several RCTs 16-26 about the use of ^125^I seeds with stent were conducted. So, we made this meta-analysis to further compare the efficacy and safety of ^125^I seeds with biliary SEMS placement versus SEMS placement alone in patients with malignant biliary obstruction.

## Materials and Methods

### Search strategy and study selection

Two reviewers (Y.X. and S.L.) independently searched Pubmed, Pubmed Central( PMC), Web of Science, ClinicalTrials.gov, Cochrane Library, Embase and CNKI databases and there were no language or geographical limitations. Articles up to May 1,2020 were figured out by key words including irradiation OR radiation OR Iodine-125 AND malignant biliary obstruction OR malignant biliary stricture OR malignant bile duct obstruction OR malignant obstructive jaundice OR malignant extrahepatic biliary obstruction AND stent. Inclusion criteria included (1)it was a RCT for unresected malignant biliary obstruction (2) stent combined with ^125^I seeds therapy was compared to stent monotherapy (3) at least one of the following was reported: survival time or rate; stent patency or stent occlusion rate. (4) full text of studies are available. (5) patients in studies did not receive other radiotherapy. Studies were excluded when information about the survival or stent patency in the patient groups was not provided. Meanwhile, non-randomized controlled trials, animal experiments, narrative reviews were excluded too.

### Data extraction and Assessment of study quality

Data were extracted by 2 authors (Y.L and Z.L) respectively based on the characteristics of studies. Detailed information included (1) basic information, such as author's last name, year and type of publication, country; (2) patient characteristics and treatment information; and (3) efficacy outcomes and adverse events. Due to the fact that all the studies were RCTs, we used the Cochrane collaboration tool to determine the risk of bias, including six items: (1) sequence generation, (2) allocation concealment, (3) blinding, (4) incomplete outcome data, (5) no selective outcome reporting, and (6) other sources of bias. For each item, a low risk counts as a score of 1, with a total of 6 scores. Any disagreement was discussed with a third author(Y, X) and resolved with our consensus.

### Statistical analysis

The primary end points were stent occlusion rates and overall survival time. Stent occlusion was dichotomous variables, so we extracted the number of events and total number of observed patients. Then, the odds ratios (OR) with 95% confidence intervals (CIs) were calculated. Also, survival was continuous variables, the means and standard deviations of observed patients were extracted from the included studies. Then, the pooled estimate of the mean difference (MD) with 95% confidence intervals (CIs) was calculated. Finally, the OR or MD of each study was pooled using either a fixed-effects or random-effects model. Moreover, when important information was not clear enough, we used digital software Engauge Digitizer (version 12.0) to extract information in the coordinates of the Kaplan-Meier curves from each of the included graphs, the survival analysis data points were extracted simultaneously. All the extracted information was exported into Microsoft Excel 2019, and then was imported into software Graphpad Prism (version 8) to analyze survival data information to reconstruct Kaplan-Meier curves and to obtain the HR (Hazard Ratio), median and mean survival time, stent occlusion rate and P value. It was considered valuable when the difference was less than 5% between the original and the extracted information. P values < 0.05 was defined as significant. A p value for Cochrane's χ^2^ <0.1 or I^2^ > 50% was considered to have high heterogeneity. Random-effects models were used when the I² >50%; otherwise, fixed-effects models were used. Publication bias was assessed by Egger's test for asymmetry. The statistical analysis was performed using Stata (version 15) and RevMan 5.3 (Cochrane Collaboration, Oxford, U.K.).

## Results

### Study selection and characteristics of included studies

**Figure 1:**
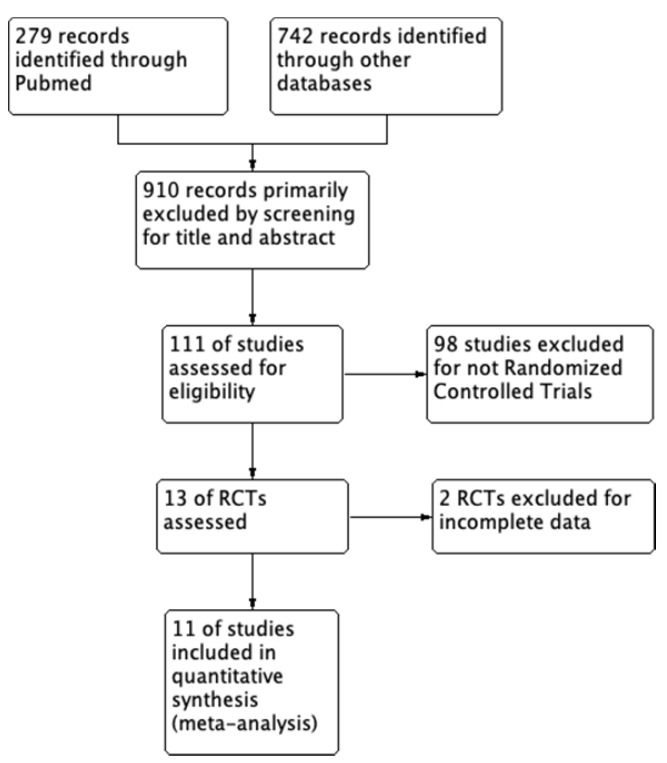
Flow program of the study enrollment process.

We strictly searched several databases for the articles based on the inclusion criteria, the number of articles was reduced from 1021 to 11 and all the included studies are RCTs (randomized controlled trials) with full text. The search process is shown in Figure 1. Within these eleven articles, a total of 767 patients were randomly distributed to either ^125^I seeds plus stent combination therapy group (377 patients) or stent monotherapy group (390 patients). Meanwhile, among these studies, all the stent implantation was performed by Percutaneous Transhepatic Cholangiography (PTC) rather than ERCP (Endoscopic retrograde Cholangiopancreatography). Technical success rate which means successful stent implantation and no shifts was almost 100%.

In addition, all the stents used in trials were ordinary metal stents like uncovered self-expandable metallic stents (USEMS) rather than plastic stents or covered self-expandable metallic stents. Moreover, seven studies [17-20, 24-26]used ^125^I seed strands and other four studies 16, 21-23 used ^125^I seed particles, which was analyzed in subgroup analysis. Detailed characteristics of studies were presented in Table 1, and basic characteristics of patients such as sex, age and etiology of malignant biliary obstruction were showed in Table 2. The Chi-square Test was conducted and all of the p value > 0.05, which means these characteristics between the two groups are well balanced.

### Stent Occlusion

**Figure 2:**
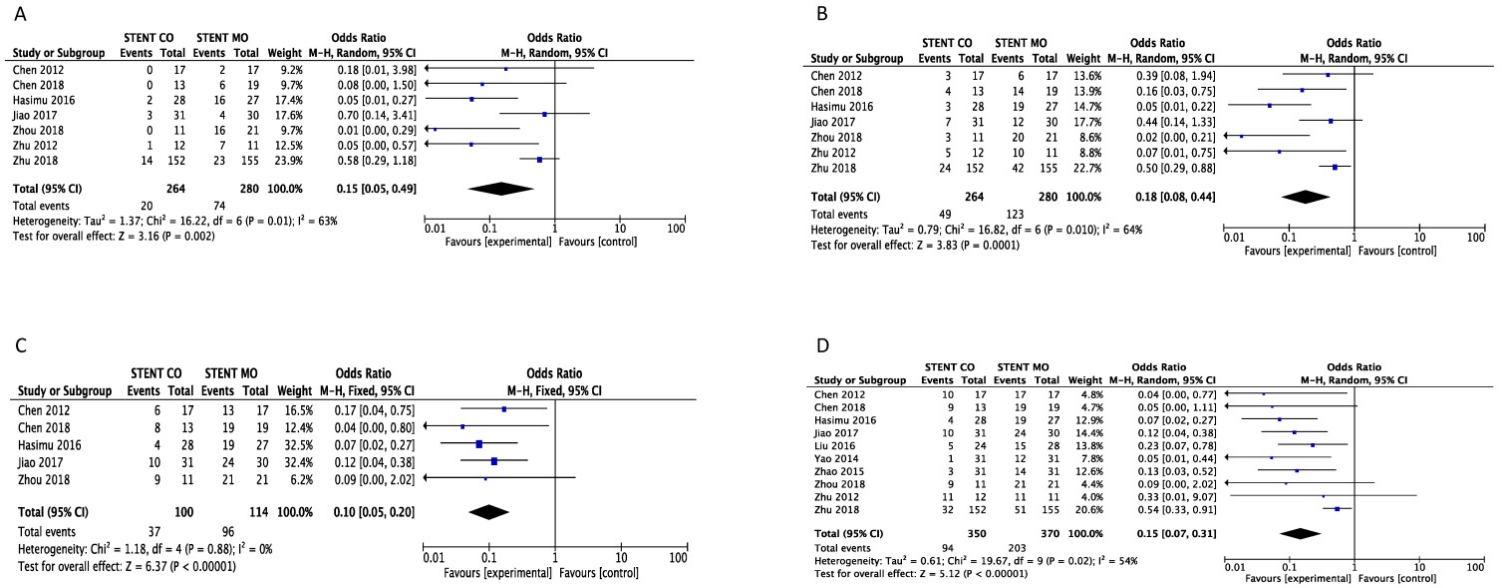
Comparison of 3-month (A), 6-month (B), 9-month(C) and 1-year (D) stent occlusion between ^125^I seeds groups and control groups. M-H: mantel-haenszel estimates; CI: confidence interval; Stent Co: stent combination therapy; Stent Mo: stent monotherapy.

Stent occlusion between the ^125^I seeds groups and control groups were measured based on different time points including 3-month 16, 20-24, 26, 6-month 16, 20-24, 26, 9-month 16, 20, 23, 24, 26 and 1-year 16, 17, 19-26. Combination therapy with ^125^I seeds demonstrated significant lower risk of stent occlusion at 3 month (OR = 0.15; 95%CI: 0.05-0.49, *P* =0.002), 6 month (OR = 0.18; 95%CI: 0.08-0.44, *P* = 0.0001), 9 month (OR = 0.10; 95%CI: 0.05-0.20, *P* < 0.00001) and 1 year (OR = 0.15; 95%CI: 0.07-0.31, *P* < 0.00001) (Figure 2). Low heterogeneity (I^2^ =0%; P = 0.88) at 9-month stent occlusion and high heterogeneity at 3-month, 1-year stent occlusion were found (I^2^ = 63%; P = 0.01, I^2^ =64%; P = 0.01, I^2^ =54%; P = 0.02 respectively) and we used random-effects models and fixed-effects models at the same time. Also, subgroup analysis and sensitivity analysis were further conducted below to find possible reasons.

### Survival

9 of 11 studies 16-18, 20-25 reported data on survival. The pooled MD was significant (MD = 125 days; 95% CI 91-159 days; P < 0.00001), suggesting that ^125^I seeds groups significantly improved mean survival compared with control groups (Figure 3A). High heterogeneity (I^2^ = 58%; P = 0.02) was found and we used random-effects models. Also, subgroup analysis and sensitivity analysis were further conducted below to find possible causes. Moreover, the pooled overall survival from reconstructed Kaplan-Meier analyses demonstrated improved survival in patients receiving Iodine-125 seeds compared with patients undergoing biliary stent placement alone (hazard ratio, 1.886; 95% CI, 1.609 to 2.210; p<0.0001) as well (Figure 3B).

### Adverse events

All the complications from studies primarily included abdominal pain, cholecystitis, pancreatitis, cholangitis and hemobilia, abdominal pain was the most common complication, but it did not reach statistical significance between ^125^I seeds + stent group and stent alone group (p=.68) and the same with the cholecystitis (p=.60), abdominal pain (p=.35), cholangitis(p=.91) and pancreatitis (*P* =.47) (Table 3).

### Subgroup analysis and Sensitivity analysis

**Figure 4:**
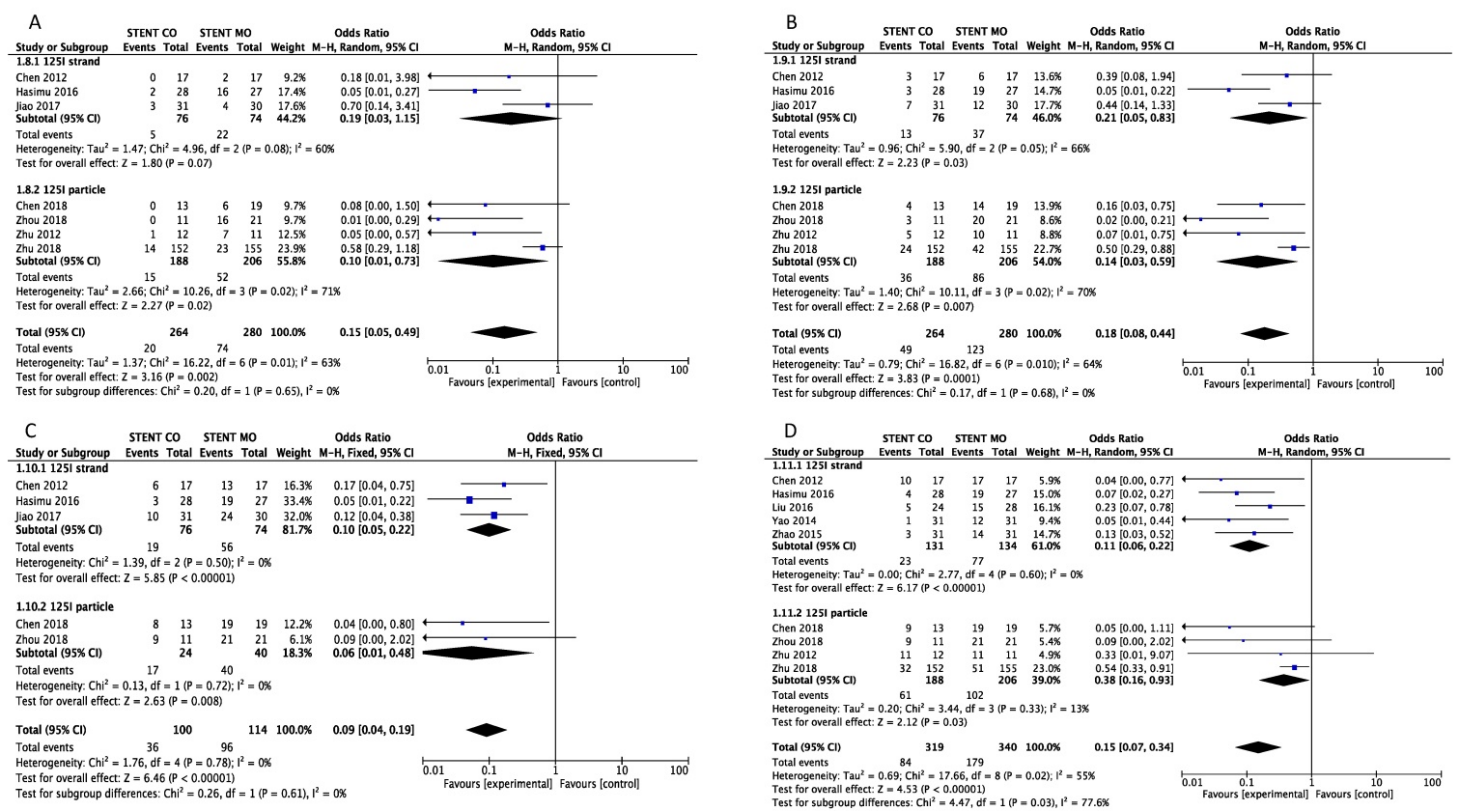
Subgroup analysis of 3-month (**A**), 6-month (**B**), 9-month(**C**) and 1-year (**D**) stent occlusion between ^125^I seeds groups and control groups.

**Figure 5:**
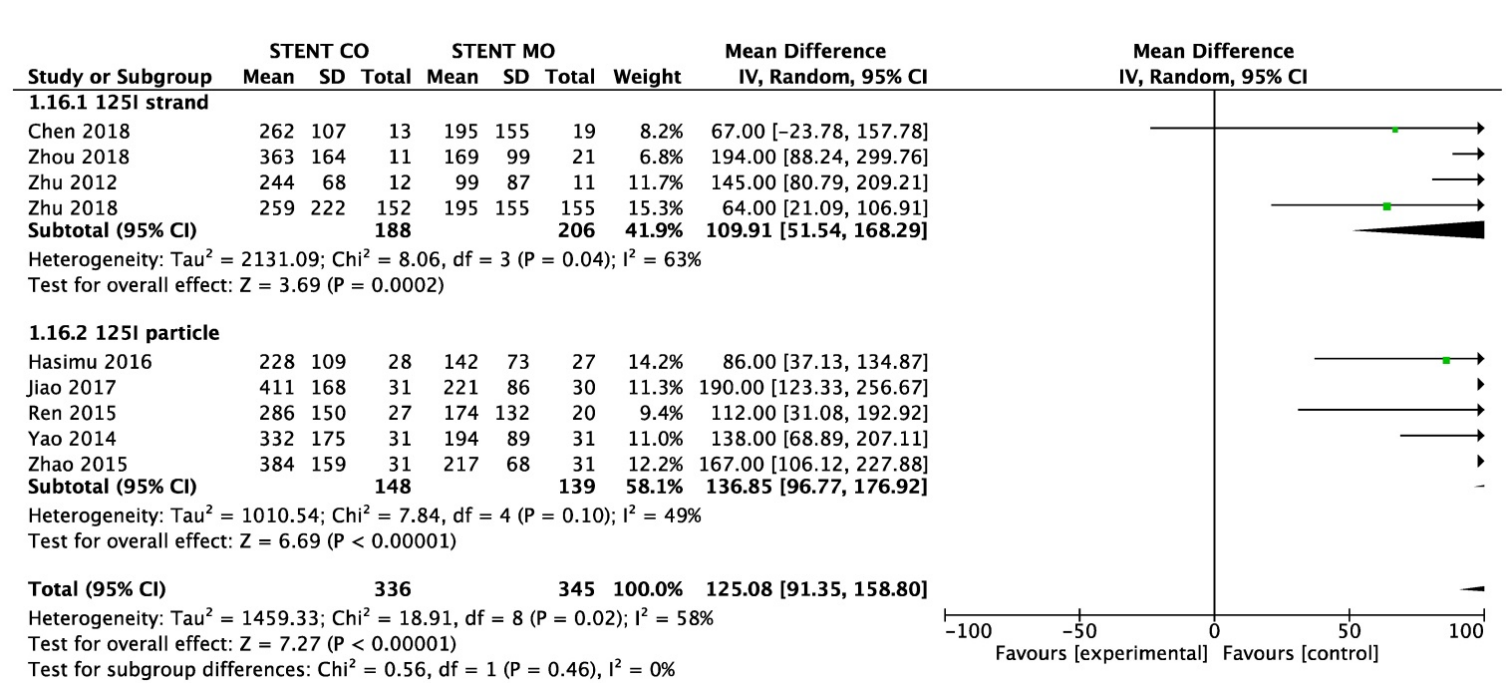
Subgroup analysis of survival between ^125^I seeds groups and control groups.

We made subgroup analysis and sensitivity analysis to find the possible reasons for high heterogeneity and intend to analyze possible factors that may influence stent occlusion and survival. We separated studies based on the different types of ^125^I seeds and seven studies [17-20, 24-26]used ^125^I seed strand and other four studies 16, 21-23 used ^125^I seed particles. Significant difference was found between ^125^I seeds groups and control groups about 6-month, 9-month and 1-year stent occlusion regardless of ^125^I seed strand or ^125^I seed particles used, but ^125^I seed strand groups showed no significant difference (p=0.07) about 3-month stent occlusion (Figure 4). Moreover, significant difference of survival between ^125^I seeds groups and control groups was found either ^125^I seed strand or ^125^I seed particles used (Figure 5). As for heterogeneity, within 1-year stent occlusion, either ^125^I seed strand group or ^125^I seed particles group showed no high heterogeneity (I^2^=0%, I^2^ =13% respectively), but the pooled result showed high heterogeneity (I^2^ =54%), which demonstrated that different types of ^125^I seeds may account for the high heterogeneity (Figure 4). However, the reason for high heterogeneity within 3-month, 6-month stent occlusion and survival were unclear, therefore, we conducted sensitivity analysis and we found Zhu et al^21^ may account for the high heterogeneity that including this study or excluding it showed huge difference. When excluded the study by Zhu 21, I^2^changed from 63% to 40%, 64% to 51%, 54% to 0% and 58% to 38% respectively at 3-month, 6-month, 1-year stent occlusion and survival (Table 4). The possible reasons were analyzed in discussion.

### Quality assessment

All the eleven trials included were from China and described proper randomization, whereas several studies provided no details of patient allocation. Only one study was blinded.

**Figure 6:**
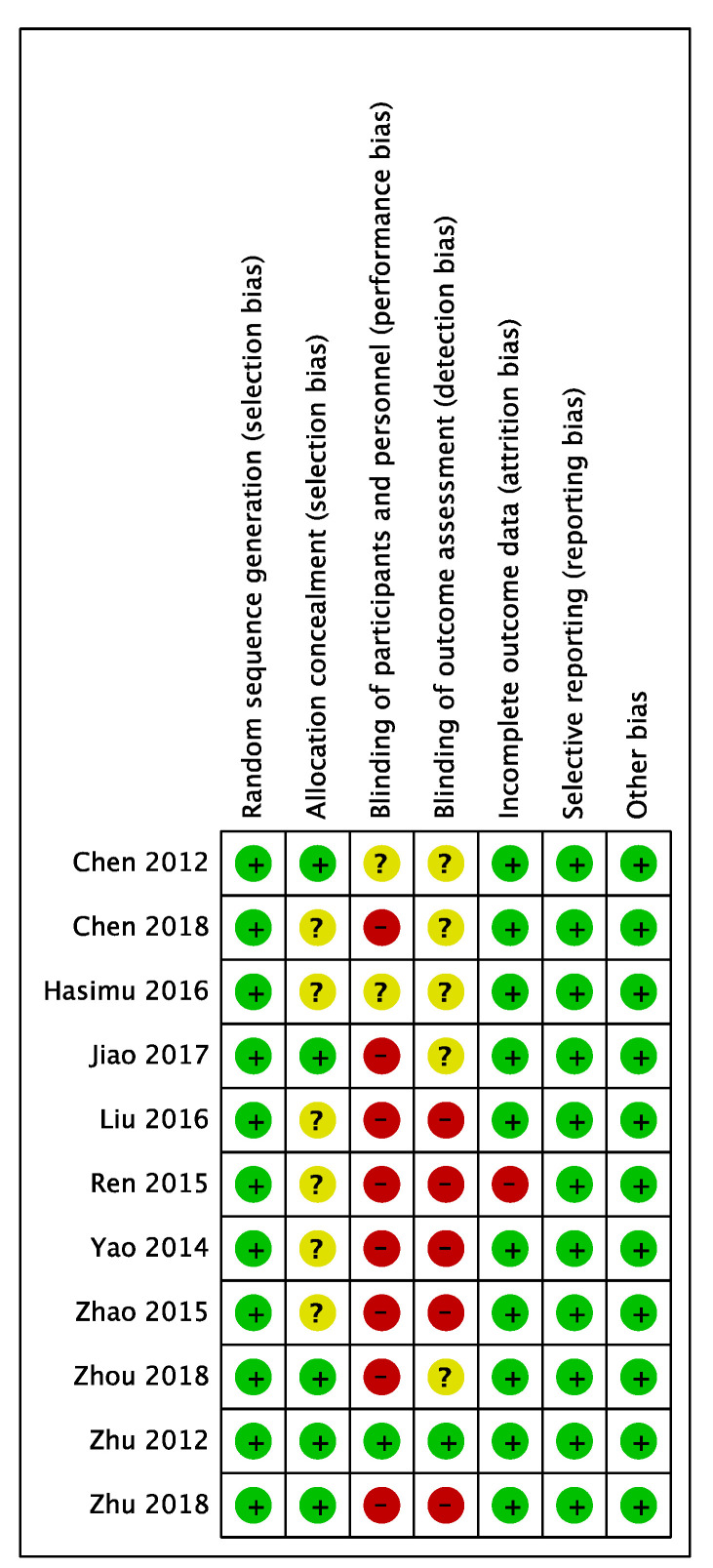
Quality of the included studies.

Validity was assessed in detail (Figure 6), the score of the trials varies from 3-7.

### Publication bias

**Figure 7:**
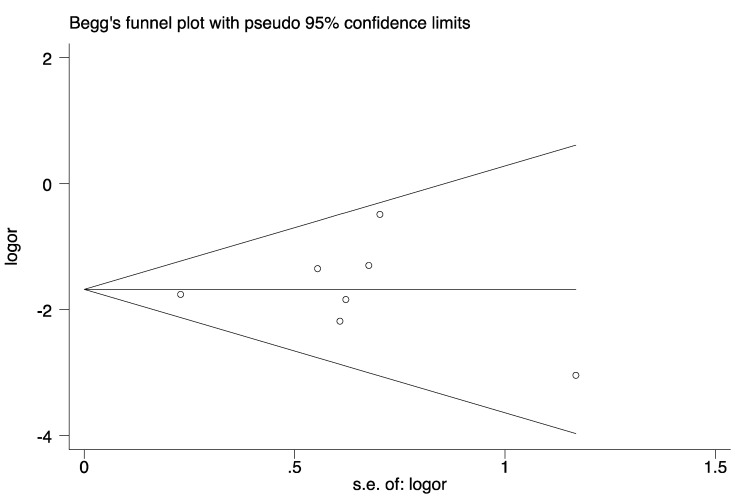
Begg's funnel plot for publication bias

Funnel plots and Egger's regression test were applied (Figure 7). Symmetrical distribution of individual studies indicated no evident publication bias. Egger: (bias=0.17; p=0.869; 95% CI:-1.99-2.88).

## Discussion

For patients with MBO who have unresectable tumors or who are unwilling to accept surgery, biliary stent placement is the preferred choice 27, 28. However, stent occlusion of patients remains challenging clinical problems. This meta-analysis of eleven RCTs 16-26 provides relatively adequate evidence in favor of ^125^I seeds combined with stent placement, which showed improved survival and lower risk of stent occlusion compared with stent placement alone. Moreover, ^125^I seeds implantation is generally safe in the treatment of patients with malignant biliary obstruction.

^125^I can emit 27.4-31.5 keV χ-rays and 35.5 keV γ-rays, with an effective radiation radius of 17-20 mm, initial dose of 7.7 cGy/h, and a radioactive half-life of 60 days and it can be kept within the tumor area to directly kill tumor cells and inhibit tumor growth into the mesh of the stent but with limited damage to surrounding normal tissues and adjacent organs and subsequently improved patients' liver function and performance status 29, 30. Furthermore, implantation of ^125^I may cause CD3^+^ and CD4^+^ cell activation, which may lead to antitumor immune responses 14. That maybe the reason why ^125^I seeds plus stent showed decreased rate of stent occlusion and superior survival.

### Subgroup analysis and sensitivity analysis

In our meta-analysis, seven studies used ^125^I seed strands and other four studies used ^125^I seed particles and we made subgroup analysis based on this difference. Although significant difference between ^125^I seeds groups and control groups was found about 6-month, 9-month and 1-year stent occlusion and survival either ^125^I seed strand or ^125^I seed particles used, even ^125^I seed strand groups showed no significant difference about 3-month stent occlusion, we speculated that ^125^I seed strand maybe the better choice. Possible reasons maybe as follows. First, irradiation dose was the focus of ^125^I seeds brachytherapy 31 and the minimum threshold for effective brachytherapy treatment of adenocarcinoma was 30 Gy, which has the capacity to kill tumor cells effectively 32. ^125^I seed strand was constructed by ^125^I seeds linearly arranged which may cumulate higher irradiation dose than ^125^I seed particles placed apart. Second, ^125^I seed strand was more easily to place with lower risk of migration than ^125^I seed particles technically because ^125^I seed strand was sealed as a single object but ^125^I seed particles had to be loaded on the stent one by one to form the irradiation stent. Third, ^125^I seed strand was easily to withdraw if radiation-related complications occurred but ^125^I seed particles could not. Last, economic payment should be considered that whether ^125^I seed strand was cheaper than ^125^I seed particles used clinically.

In addition, based on ^125^I seeds plus stent placement, whether different etiology of malignant biliary obstruction such as cholangiocarcinoma, pancreatic cancer or hepatocellular carcinoma had effects on survival of patients, whether different types of stent influenced the stent patency, whether extrahepatic and intrahepatic biliary obstruction showed different therapeutic prognosis, whether ^125^I seeds plus stent placement is superior to RFA combined with stent, ought to be analyzed. However, there was not enough information in the included studies and more studies should be conducted to figure out the precise and specialized treatment for patient with MBO.

Furthermore, we conducted sensitivity analysis and found that high heterogeneity of 3-month, 6-month and 1-year stent occlusion disappeared when the study by Zhu 21 was excluded. Possible reasons maybe as follows. First, number of patients in this study were large that almost occupied the half of the total number. Second, some patients who underwent stent occlusion received stent implantation again in this study. Third, some patients received chemotherapy after stent placement too.

### Advantages and disadvantages

In our meta-analysis, we included eleven RCTs which provided relatively adequate evidence and we used digital software Engauge Digitizer to reconstruct Kaplan-Meier curves and obtain the HR, median and mean survival time as well. However, there were several disadvantages in our meta-analysis. First, high heterogeneity was found in 3-month, 6-month and 1-year stent occlusion and we performed subgroup analysis and sensitivity analysis to find possible causes. Second, several included studies were of high quality that complied with the rules of RCT strictly but several studies did not abide by the rules strictly enough. Third, all the 11 studies were conducted in China, which may diminish the adequate representation of the current study. Last, sample size maybe not huge enough and included patients had various kinds of cancers which may hinder the applicability of this analysis.

In conclusion, ^125^I seeds plus stent placement was significantly superior to stent placement alone with regard to stent patency and survival of patients. In addition, ^125^I seeds implantation was a safe and tolerable procedure with comparable complications to stent placement alone. It may be a promising therapy for patients with malignant biliary obstruction and more well-designed randomized trials are necessary to further confirm these conclusions.

## Author Contributions

Y.X. and W.W. conceived the idea. Y.X. and S.L. searched and included articles and wrote the manuscript text. Y.L. and Z.L. collected and analyzed the data. Y.X. and Y.L. reconciled disagreements. S.L. and Z.L. provided statistic support. All authors reviewed the manuscript.

## Figures and Tables

**Figure 3A:**
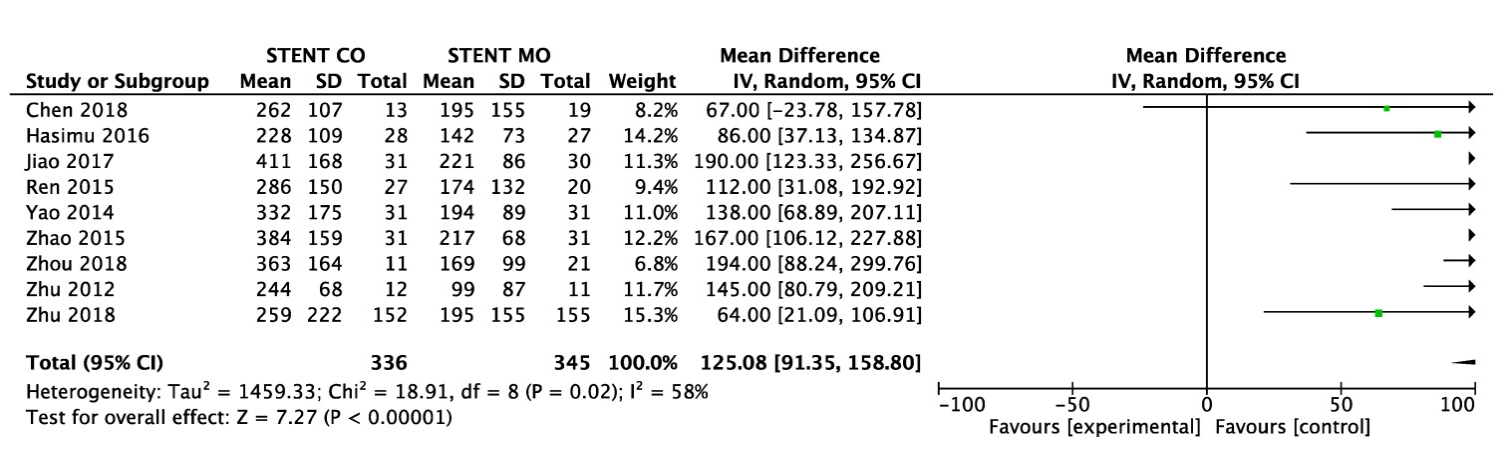
Comparison of survival between ^125^I seeds groups and control groups.

**Figure 3B:**
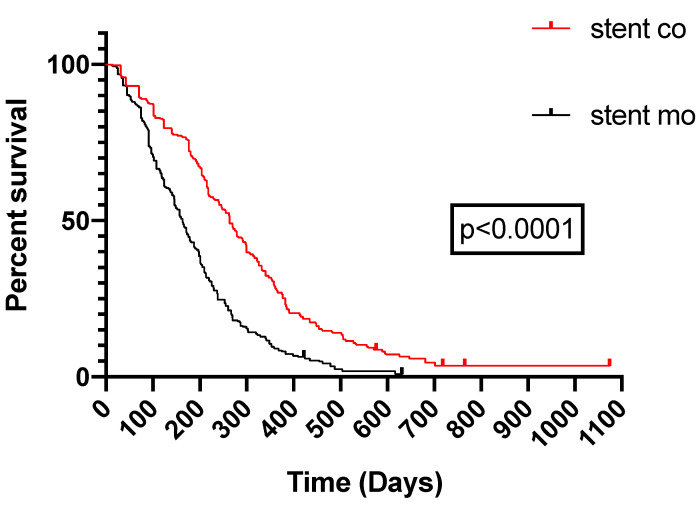
Pooled Kaplan-Meier survival analysis of ^125^I seeds groups and control groups.

**Table 1:** Basic characteristics of the included trials

Author (year)	Country	Period	Study design	Article type	Treatment			
					Intervention		Control	
					No. of patients	Stent type	No. of patients	Stent type
Hasimu (2016)	China	July2011-June 2014	RCT	Full text	28	Stent with ^125^I seed strand	27	A nitinol self-expandable stent
Zhu (2012)	China	November2008-October 2010	RCT	Full text	12	An outer self-expandable ^125^I seeds-loaded- stent and an inner conventional self-expanding biliary nitinol alloy stent	11	Self-expanding biliary nitinol alloy stent
Jiao (2017)	China	January2013-January 2015	RCT	Full text	31	Stent with ^125^I seed strand	30	A Nitinol self-expandable stent
Zhu (2017)	China	October2013-March2016	RCT	Full text	152	An inner conventional uncovered SEMS and an outer ^125^I seeds-loaded stent	155	Uncovered SEMS
Chen (2012)	China	March2009-January 2010	RCT	Full text	17	^125^I seed strand with stent	17	A Nitinol self-expandable stent
Chen(2018)	China	September2014-November2016	RCT	Full text	13	^125^I seed particle stent particle	19	Ordinary metal stent
Zhou (2018)	China	May2015-September2016	RCT	Full text	11	Iodine-125 seed particle	21	Uncovered SEMS
Yao (2014)	China	February2012-April2014	RCT	Full text	31	^125^I seed strand with stent	31	Uncovered SEMS
Liu (2016)	China	October2012-March2015	RCT	Full text	24	^125^I seed strand with stent	28	Uncovered SEMS
Zhao (2015)	China	July2013-January2015	RCT	Full text	31	^125^I seed strand with stent	31	Uncovered SEMS
Ren (2015)	China	March2012-February2013	RCT	Full text	27	^125^I seed strand with stent	20	Uncovered SEMS

RCT: randomized controlled trial; 125I: iodine-125; SEMS: self-expandable metal stent.

**Table 2:** Basic characteristics of the included patients

Ref	Intervention	Patient (no.)	Mean age (year)	Mean length of stricture(mm)	Sex male no.(%)	Etiology of MBO
Hasimu (2016)	^125^I group Stent group	28 27	70.93(8.68) 70.26(9.71)	60.89(9.84) 61.78(8.12)	11(39.3) 14(51.9)	mixed mixed
Zhu (2012)	^125^I group Stent group	12 11	62.5(17) 71(18)	42.5(7.2) 40(5.7)	7(58.3) 9(81.8)	mixed mixed
Jiao (2017)	^125^I group Stent group	31 30	60.4(8.8) 60.2(8.1)	29.5(6.8) 30.1(7.5)	12(41.4) 16(53.3)	mixed mixed
Zhu (2017)	^125^I group Stent group	152 155	65(14) 65(13)	35(7.5) 32(11.2)	103(67.8) 109(70.3)	mixed mixed
Chen (2012)	^125^I group Stent group	17 17	61.2(14.5) 63.9(9.3)	NA NA	12(70.6) 10(58.8)	mixed mixed
Chen (2018)	^125^I group Stent group	13 19	66 68	NA NA	8(61.5) 12(63.2)	mixed mixed
Zhou (2018)	^125^I group Stent group	11 21	NA NA	NA NA	7(63.3) 13(61.9)	mixed mixed
Yao (2014)	^125^I group Stent group	31 31	NA NA	NA NA	NA NA	mixed mixed
Liu (2016)	^125^I group Stent group	24 28	NA NA	NA NA	NA NA	mixed mixed
Zhao (2015)	^125^I group Stent group	31 31	68(3.5) 68(3.5)	NA NA	28(45)	mixed mixed
Ren (2015)	^125^I group Stent group	27 20	52(5.3) 52(5.3)	NA NA	32(68)	mixed mixed

MBO: malignant biliary obstruction; Mixed: various kinds of cancers.

**Table 3:** Adverse events in ^125^I seeds group and control group

	^125^I + stent (n=377)	Stent only (n=390)	P value
Abdominal pain	4.7	4.1	0.68
Hemobilia	2.2	2.1	0.99
Pancreatitis	1.1	0.7	0.47
Cholangitis	3.4	2.6	0.91
Cholecystitis	0.8	1.2	0.60

Values are percentages.

**Table 4:** Outcome of sensitivity analysis

3-month stent occlusion	I^2^	OR and 95%CI	P value
Include Zhu(2018)	63%	0.15[0.05,0.49]	=0.002
Exclude Zhu(2018)	40%	0.11[0.05,0.24]	<0.00001
**6-month stent occlusion**	**I^2^**	**OR and 95%CI**	**P value**
Include Zhu(2018)	64%	0.18[0.08,0.44]	=0.0001
Exclude Zhu(2018)	51%	0.14[0.05,0.36]	<0.0001
**12-month stent occlusion**	**I^2^**	**OR and 95%CI**	**P value**
Include Zhu(2018)	54%	0.15[0.07,0.31]	<0.00001
Exclude Zhu(2018)	0%	0.16[0.06,0.20]	<0.00001
**Overall survival**	**I^2^**	**MD and 95%CI**	**P value**
Include Zhu(2018)	58%	125[91,159]	<0.00001
Exclude Zhu(2018)	38%	133[109,157]	<0.00001

## Abbreviations

MBO: malignant biliary obstruction
RCT: randomized controlled trial
^125^I: iodine-125
SEMS: self-expandable metal stent
PTC: percutaneous transhepatic cholangiography
ERCP: endoscopic retrograde cholangiopancreatography
OR: odds ratios
MD: mean difference
CI: confidence interval
